# Diabetes and Risks of Right-Sided and Left-Sided Colon Cancer: A Meta-Analysis of Prospective Cohorts

**DOI:** 10.3389/fonc.2022.737330

**Published:** 2022-04-07

**Authors:** Wenxuan Xiao, Jinglong Huang, Chuanyi Zhao, Lu Ding, Xuan Wang, Bian Wu

**Affiliations:** ^1^ Cancer Center, Union Hospital, Tongji Medical College, Huazhong University of Science and Technology, Wuhan, China; ^2^ Department of Neurosurgery, Union Hospital, Tongji Medical College, Huazhong University of Science and Technology, Wuhan, China

**Keywords:** colon cancer, diabetes, risk factors, meta-analysis, prospective cohort studies

## Abstract

**Background and Aims:**

Diabetes is associated with an increased risk of colon cancer (CC). Epidemiologic studies previously reported a higher risk for right-sided colon cancer (RCC) compare to left-sided colon cancer (LCC), although data are conflicting. We performed a meta-analysis to investigate this issue.

**Methods:**

We systematically searched the PubMed, EMBASE, Web of Science and Cochrane Library database for prospective cohort studies published up to June 2021. Studies were included if they reported site-specific estimates of the relative risk (RR) between diabetes and the risks of RCC and LCC. Random effects meta-analyses with inverse variance weighting were used to estimate the pooled site-specific RRs and the RCC-to-LCC ratio of RRs (RRRs).

**Results:**

Data from 10 prospective cohort studies, representing 1,642,823 individuals (mainly white) and 17,624 CC patients, were included in the analysis. Diabetes was associated with an increased risk of both RCC (RR =1.35, 95% CI = 1.24-1.47) and LCC (RR = 1.18, 95% CI = 1.08-1.28). After adjusting for major risk factors, individuals with diabetes had a greater risk for RCC than for LCC (RRR = 1.13, 95% CI = 1.02-1.26), with no significant heterogeneity between studies (I^2^ = 0%).

**Conclusions:**

This meta-analysis indicates that diabetes is associated with a higher risk for RCC than for LCC. Our findings suggest that colonoscopic surveillance in diabetic patients with careful examination of the right colon is warranted.

## Introduction

The relationship between diabetes and the risk of colon cancer (CC), two highly prevalent and major health problems worldwide, is well recognized. Most previous epidemiologic studies have provided evidence that individuals with diabetes have an increased risk of CC compared with their nondiabetic counterparts (1–4). An updated 2011 meta-analysis suggested that diabetes was an independent risk factor for colon and rectal cancer, after adjusting major risk factors including obesity, smoking and physical activity, with a corresponding summary relative risk (RR) of 1.38 and 1.20, respectively (5). The so-called “hyperinsulinemia hypothesis”, which suggests that elevated levels of insulin and insulin-like growth factor-1 (IGF-1) increase the risk of CC by promoting the growth of colon cells and acting as a cell mitogen, provides the underlying mechanism for this connection (6).

Although extensive research has been performed on this topic, several features of the association between diabetes and the risk of CC remain unclear. For instance, it is not known whether diabetes is differentially associated with the risks of right-sided colon cancer (RCC) and left-sided colon cancer (LCC). A growing body of evidence suggests that LCC and RCC should be considered two distinct clinical and biological tumor entities. The left side of the colon originates from the midgut, whereas the right side originates from the hindgut. Differences also exist that correlate to physical function, artery supply, histology and biochemical features (7, 8). Subsequent research has shown that there are distinct differences in epidemiology (9), pathogenesis, genetic landscape, molecular pathways (10), and the clinical outcome (11, 12) between cancers at these two anatomical sites.

Understanding whether diabetes is differentially associated with the risks of developing RCC and LCC may have important clinical and public health implications. Screening for colorectal cancer (CRC) in average-risk or high-risk patients using flexible sigmoidoscopy (FS) or colonoscopy is now common in many countries. However, FS screens only the distal colon (10). In 2005, Limburg and colleagues first reported that diabetes was significantly associated with the risk of RCC, but not LCC, and suggested that CRC screening methods should include evaluation of the right colon for patients with diabetes to improve the effectiveness of CRC prevention (13). However, findings from other previous studies have been inconsistent (1, 14), and there has been no systematic comparison of subsite differences between diabetes and CC risk.

Given the rising prevalence of diabetes as a global health problem and the substantial clinical implications that any important subsite difference in the association between diabetes and CC risk would have, we undertook a meta-analysis of all available prospective cohort studies that reported the site-specific effects of diabetes on the subsequent risk of CC.

## Methods

This study was conducted according to the Meta-analysis Of Observational Studies in Epidemiology (MOOSE) guidelines (15).

### Search Strategy

A comprehensive, computerized literature search was performed using the PubMed, Embase, Web of Science and Cochrane Library database up until June, 2021. The following search key words were used: “diabetes”, “diabetes mellitus”, “colorectal”, “colorectal”, “colon”, “neoplasm”, “cancer”, “carcinoma” and “tumor”. Details on the search strategy are provided in the **Supplementary Methods**. Reference lists of relevant articles were hand searched for potentially eligible studies.

### Study Selection

Studies included in our meta-analysis were required to meet the following inclusion criteria: 1) used a prospective cohort design; 2) reported the RR and the corresponding 95% confidence interval (CI); 3) classified CC into no more than two outcomes (i.e., right/left side CC, proximal/distal CC); 4) defined the proximal or right-sided colon as including at least the cecum, the ascending colon, and the transverse colon, but no anatomical sites distal to the splenic flexure, and the distal or left-sided colon as including at least the descending and sigmoid colons, but not the rectosigmoid junction or the rectum, and no anatomical sites proximal to the splenic flexure; 5) included only the most recent publication when duplicate reports from the same study were identified; and 6) classified at least 80% of CRC cases or CC cases by subsite. Meeting abstracts, commentaries and letters were excluded.

### Data Extraction

Data were extracted independently by two authors, and discrepancies were resolved by team consensus. The following information was extracted from each eligible study and entered into a structured database: study name, year of publication, country, age (mean and range), sample size, prevalence of diabetes, the numbers of RCC and LCC, year of baseline data collection, study duration, RRs reflecting the greatest degree of control for potential confounders were adopted in the meta-analysis. We also contacted the corresponding author *via* email if a study reported insufficient data (i.e., RRs and 95% CIs) to include in the meta-analysis. For studies that provided separate RRs for men and women, we pooled the RRs weighted by the inverse of the variance within each study.

### Assessment of the Risk of Bias in Individual Studies

The quality of the included studies was evaluated using the Newcastle-Ottawa Scale (NOS) (11). This scale assessed the likelihood of bias in 3 parts: (1) selection of the study groups; (2) comparability of groups; and (3) ascertainment of exposure and outcome. Studies with a cumulative score ≥ 7 were considered to have a low risk of bias, scores of 4 to 6 as having a moderate risk of bias, and scores less than 4 as having a high risk of bias. Concerning whether the follow-up was sufficient for outcomes to occur, we set the minimum follow-up to 10 years.

### Statistical Analysis

For each study, we extracted the site-specific RRs and 95% CIs for individuals with diabetes versus individuals without diabetes. Adjusted RRs were used for the analysis to account for confounding variables. If a study reported results for males and females separately, we estimated the pooled RRs. These RRs and 95% CIs were subsequently used to calculate the RCC-to-LCC ratio of RRs (RRRs) and 95% CIs, which compared the association between diabetes and the risk of RCC with the association between diabetes and the risk of LCC (16, 17).

We used the random effects model described by DerSimonian and Laird to estimate the pooled RRs and 95% CIs with inverse-variance weighting (18). An identical approach was used for the RRRs. We calculated the standard error of the log RRR by taking the square root of the sum of the variance of the two site-specific log RRs for each study. Heterogeneity between individual studies was assessed by Cochran’s Q statistic and the I^2^ statistic; p ≤ 0.05 or I^2^ > 50% indicated significant heterogeneity (19).

We conducted sensitivity analyses by geographical area, sex, duration of follow-up, different definitions of RCC and LCC used in the study (studies that included the splenic flexure as part of the right colon were classified as having used definition 1; studies that included the splenic flexure as part of the left colon were classified as having used definition 2) and level of adjustment. We use random effects meta-regression analyses to examine whether there was a significant difference between the subgroups and whether differences in the duration of the study follow-up and prevalence of diabetes contributed to heterogeneity between studies.

We investigated publication bias by visual inspection of funnel plots, Begg’s rank correlation test and Egger’s regression test (20, 21). All statistical tests were two-sided, with a p value < 0.05 considered significant for all tests. The statistical analysis was independently performed by two authors (W.X. and J.H.), using Stata software (version 15.1; Stata Corporation, College Station, Texas, USA). Disagreements were again resolved by team consensus.

## Results

### Study Selection

**Figure 1:**
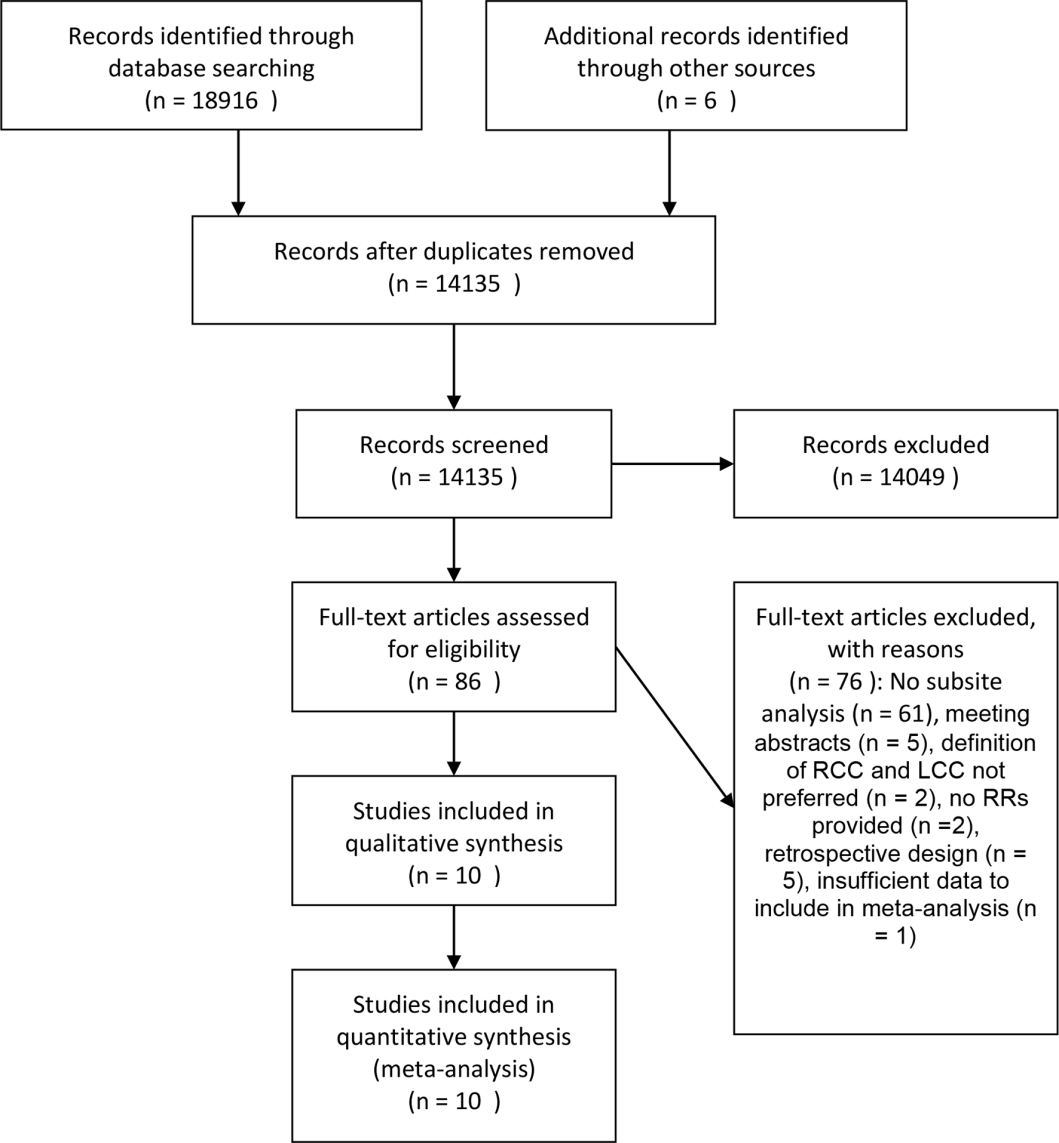
Flow chart of study selection.

The detailed steps of the systemic research are shown in **Figure 1**. In brief, we identified 14135 unique articles. After the screening of the titles and abstracts, a total of 86 cohort studies that investigated the association between diabetes and the risk of CC or CRC remained. After a full-text assessment, 61 studies that did not report RRs by colonic subsites and five studies that were meeting abstracts were excluded. Other studies were excluded for various reasons: two studies did not use RRs to evaluate the results (22, 23); two studies did not meet the required definition of RCC and LCC (24, 25); five studies were used a retrospective design (26–30); and one study was excluded because 71% of the CC cases could not be classified by location (31). The remaining ten studies fulfilled our inclusion criteria and were included in the meta-analysis (1, 2, 13, 32–38).

### Study Characteristics

The characteristics of these studies are shown in **Table 1**. All studies were prospective cohort studies. The baseline survey ranged from 1976 to 2003, and the duration of follow-up was between 7 to 20.3 years (median: 15 years). Overall, data were available from 1,642,823 individuals, of whom 17,624 had CC. Six of the studies were conducted in the USA, one in Norway, one in Sweden, one in the Netherlands and one in 10 European countries (Denmark, France, Germany, Greece, Italy, the Netherlands, Norway, Spain, Sweden, and the United Kingdom). Eight studies used definition 1 to classify the anatomical sites as left-sided and right-sided colon cancer, and two studies used definition 2. Three studies involved both males and females and reported sex-specific results; three studies involved both males and females and did not report sex-specific results; and two studies involved males only and two involved females only. The prevalence of diabetes ranged from 2.8 to 15.7%. Nine studies provided multi-adjusted RRs and five studies provided both age-adjusted and multi-adjusted RRs.

**Table 1:** Characteristics of included studies.

Study name, year	Country	Age range (mean age)	Sample size	Prevalence of diabetes	RCC cases	LCC cases	Year of baseline data collection	Study duration (years)	Diabetes assessment	Definition type	Adjusted variables
**Nurses’ Health Study (NHS) (** 1 **), 1999**	USA	30–55 (42.2)	118,072	6%	275	332	1976	18	Self-report questionnaire/medical records	1^a^	Age, time periods, BMI, smoking, menopausal status, multivitamin supplement, alcohol, physical activity, aspirin, parental history of CRC and red meat
**Cohort of Swedish men (COSM) (** 2 **), 2005**	Sweden	45–79 (N/A)	45,550	6.2%	98	92	1997	7	Self-report questionnaire	1	Age, BMI, education, family history of CRC, physical activity, smoking, multivitamin supplement, aspirin, consumption of fruits, vegetables, dairy foods and red meat
**Iowa Women’s Health Study (IWHS) (** 13 **), 2005**	USA	55–69 (61.5)	34,972	5.4%	402	259	1986	14	Self-report questionnaire	1	Age, BMI, total energy intake, calcium intake, vitamin E intake
**Physician’s Health Study (PHS) (** 38 **), 2006**	USA	40–84 (53.8)	22,046	8.6%	192	151	1982	21	Self-report questionnaire	1	Age, vigorous exercise, smoking, alcohol, multivitamin use, NSAID use, arthritis, and consumption of fruits and vegetables
**Cancer Prevention Study II Nutrition Cohort (CPS-II) (** 32 **), 2010**	USA	50-74 (63.0)	154,975	7.3%	N/A	N/A	1992-1993	15	Self-report questionnaire /Medical records	1	Age, education, body mass index, physical activity, NSAID use, alcohol use, family history of CRC
**Multiethnic Cohort (MEC) (** 34 **), 2010**	USA	45–75 (59.9)	199142	15.7%	1464	1091	1993-1996	13	Self-report questionnaires	1	Age, entry of the cohort, race, BMI, smoking, NSAIDs, education, alcohol, (un-)saturated fat intake, dietary fiber, physical activity and family history of CRC
**National Institute of Health-AARP Diet and Health Study (NIH-AARP) (** 35 **), 2013**	USA	50-71 (62.0)	484,020	8.6%	3,063	2,229	1995-1996	11.2	Self-report questionnaires	2^b^	Age, race/ethnicity, education, BMI, smoking, physical activity, replacement hormone therapy in women, family history of colon cancer and vitamin and mineral supplements
**Cohort of Norway (CONOR) (** 36 **), 2015**	Norway	N/A (50.9)	143,477	3.1%	853	606	1994-2003	15	Self-report questionnaires	1	Age, sex, smoking, alcohol consumption, physical activity, education, family history of cancer, and BMI
**Netherlands Cohort Study on diet and cancer (NLCS) (** 33 **), 2016**	Netherlands	55-69 (N/A)	114,503	3.7%	1,614	1,421	1986	20.3	Self-report questionnaires	2	Age
**European Prospective Investigation int o Cancer and Nutrition study (EPIC) (** 37 **), 2019**	European countries (Denmark, France, Germany, Greece, Italy, the Netherlands, Norway, Spain, Sweden, and the United Kingdom)	N/A (51.3)	476,160	2.8%	1,877	1,743	1992-2000	14.9	Self-report questionnaire	1	Age, body mass index, height, physical activity index; smoking status and intensity; education level attained; ever use of menopausal hormone therapy; and intakes of alcohol, red and processed meats, dietary calcium, and fiber

RR, relative risk; CI, confidence interval; N/A, not available; RCC, right-sided colon cancer; LCC, left-sided colon cancer; BMI, body mass index; CRC, colorectal cancer; NSAIDs, nonsteroidal anti-inflammatory drugs.

a Definition 1 = splenic flexure included as part of the right colon.

b Definition 2 = splenic flexure included as part of the left colon.

### Quality Assessment of the Risk of Bias of the Included Studies

Rating of the quality of the included studies according to the NOS is presented in **Supplementary Table 1**. All of the ten studies were considered to have a low risk of bias. All studies included a control group from the same community as the exposed group. Most studies adjusted for the following confounders: age, body mass index (BMI), smoking, physical activity, alcohol intake, non-steroidal anti-inflammatory drug (NSAID) intake, multivitamin use, and family history of CRC.

Relative Risk Between Diabetes and the Risks of Right-Sided Colon Cancer and Left-Sided Colon Cancer

**Figure 2:**
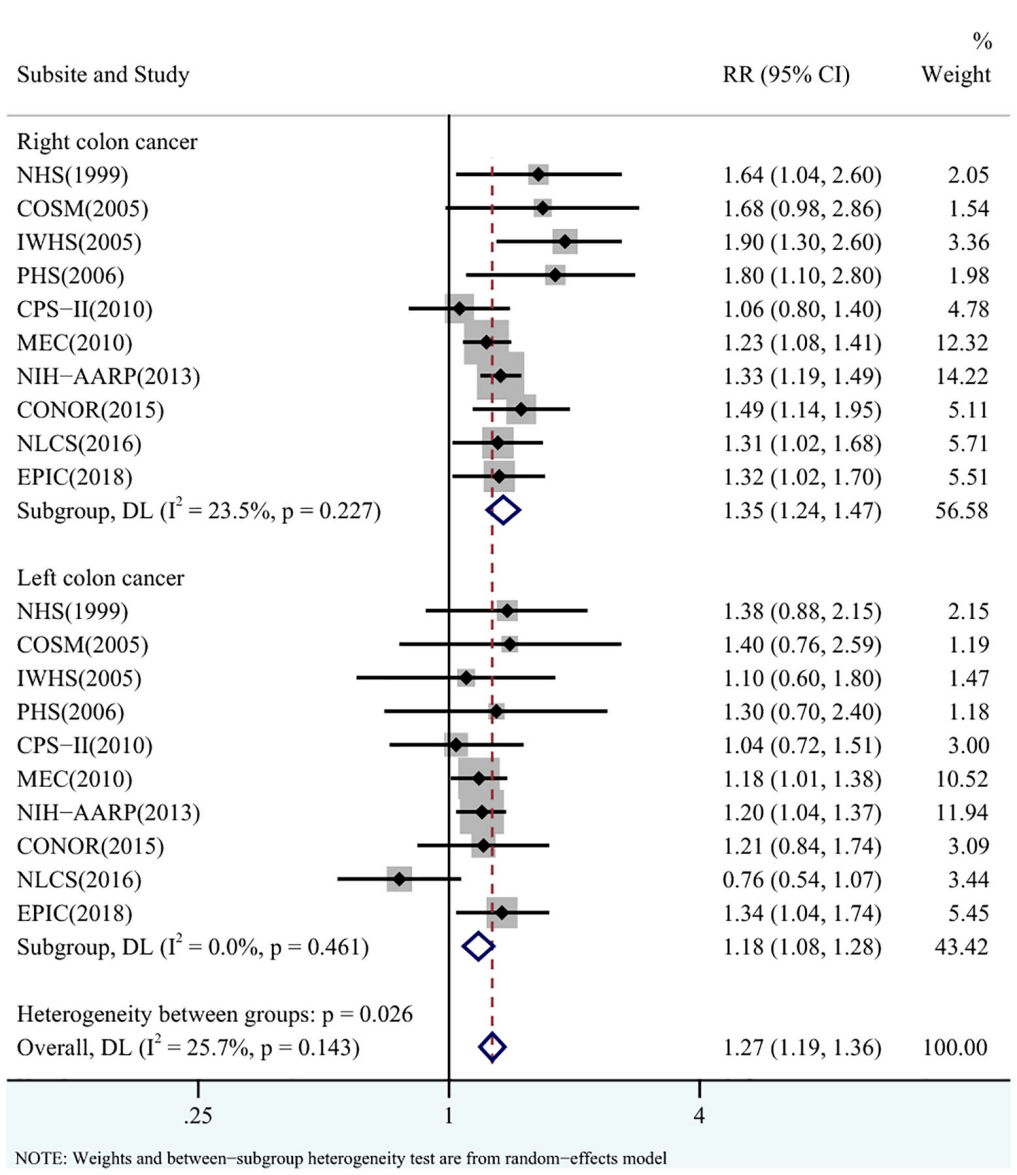
Pooled relative risks (RRs) for the association between diabetes and the risks of right-sided colon cancer (RCC) and left-sided colon cancer (LCC).

In general, diabetes was associated with an increased risk of CC compared with no diabetes (RR = 1.27, 95% CI = 1.19-1.36), with low heterogeneity between studies (I^2^ = 25.7%, p = 0.143) (**Figure 2**).

Diabetes was associated with an increased risk of RCC compared with no diabetes (RR = 1.35, 95% CI = 1.24-1.47), with low heterogeneity between studies (I^2^ = 23.5%, p = 0.227) (**Figure 2**). There was no evidence of publication bias (Begg’s test: p = 0.107, Egger’s test: p = 0.090; **Supplementary Figure S1**). In the sensitivity analyses, the pooled RR did not vary materially by geographical area (p = 0.837), sex (p = 0.600), definition of RCC and LCC (p = 0.548), duration of follow-up (p = 0.904) or level of adjustment (p = 0.947) (**Table 2**).

**Table 2:** Relative risks (RRs) and 95% confidence intervals (CIs) for sensitivity analysis.

	No. of studies	CC (colon cancer)				RCC (right-sided colon cancer)				LCC (left-sided colon cancer)			
		RR (95%CI)	I^2^,%	*P*_heterogeneity_	*P* value for interaction	RR (95%CI)	I^2^,%	*P*_heterogeneity_	*P* value for interaction	RR (95%CI)	I^2^,%	*P*_heterogeneity_	*P* value for interaction
**Geographical area**													
** USA**	6	1.29 (1.16-1.42)	43.5	0.115	0.156	1.36 (1.18-1.56)	51.2	0.069	0.837	1.19 (1.08-1.31)	0	0.953	0.732
** Europe**	4	1.28 (1.12-1.47)	29.7	0.234		1.39 (1.20-1.60)	0	0.774		1.13 (0.84-1.51)	59.7	0.059	
**Sex**													
** Males**	5	1.24 (1.10-1.40)	32.5	0.205	0.025	1.30 (1.17-1.45)	0	0.445	0.600	1.15 (1.01-1.31)	0	0.444	0.682
** Females**	5	1.28 (1.05-1.57)	68.6	0.013		1.39 (1.10-1.77)	66.5	0.018		1.09 (0.87-1.37)	30.6	0.217	
**Definition of RCC and LCC**													
** Definition 1**	8	1.32 (1.20-1.46)	33.4	0.162	0.156	1.40 (1.22-1.60)	40.4	0.109	0.548	1.21 (1.09-1.35)	0	0.957	0.362
** Definition 2**	2	1.21 (1.03-1.41)	56.9	0.128		1.33 (1.20-1.47)	0	0.914		0.98 (0.63-1.53)	83.0	0.015	
**Duration of follow-up**													
** < 15 years**	5	1.29 (1.20-1.38)	16.3	0.311	0.156	1.35 (1.21-1.51)	34.5	0.191	0.904	1.21 (1.10-1.33)	0	0.902	0.321
** ≥ 15 years**	5	1.26 (1.07-1.48)	49.9	0.092		1.37 (1.15-1.62)	28.5	0.232		1.07 (0.86-1.34)	32.4	0.206	
**Level of adjustment**													
**Age-adjusted**	5	1.30 (1.15-1.47)	31.8	0.209	0.175	1.33 (1.15-1.54)	22.2	0.273	0.947	1.24 (1.10-1.40)	0	0.799	0.685
** Multi-adjusted**	5	1.28 (1.11-1.48)	39.6	0.157		1.32 (1.11-1.58)	35.5	0.184		1.19 (1.05-1.36)	0	0.863	

Diabetes was also associated with an increased risk of LCC (RR = 1.18, 95% CI = 1.08-1.28), with no heterogeneity between studies (I^2^ = 0%, p = 0.461) (**Figure 2**) and no evidence of publication bias (Begg’s test: p = 1.000, Egger’s test: p = 0.827; **Supplementary Figure S2**). In the sensitivity analyses, there was no evidence that the pooled RR differed significantly by geographical area (p = 0.732), sex (p = 0.682), definition of RCC and LCC (p = 0.362), duration of follow-up (p = 0.321) or the level of adjustment (p = 0.685) (**Table 2**).

### RCC-to-LCC Ratio of Relative Risk

**Figure 3:**
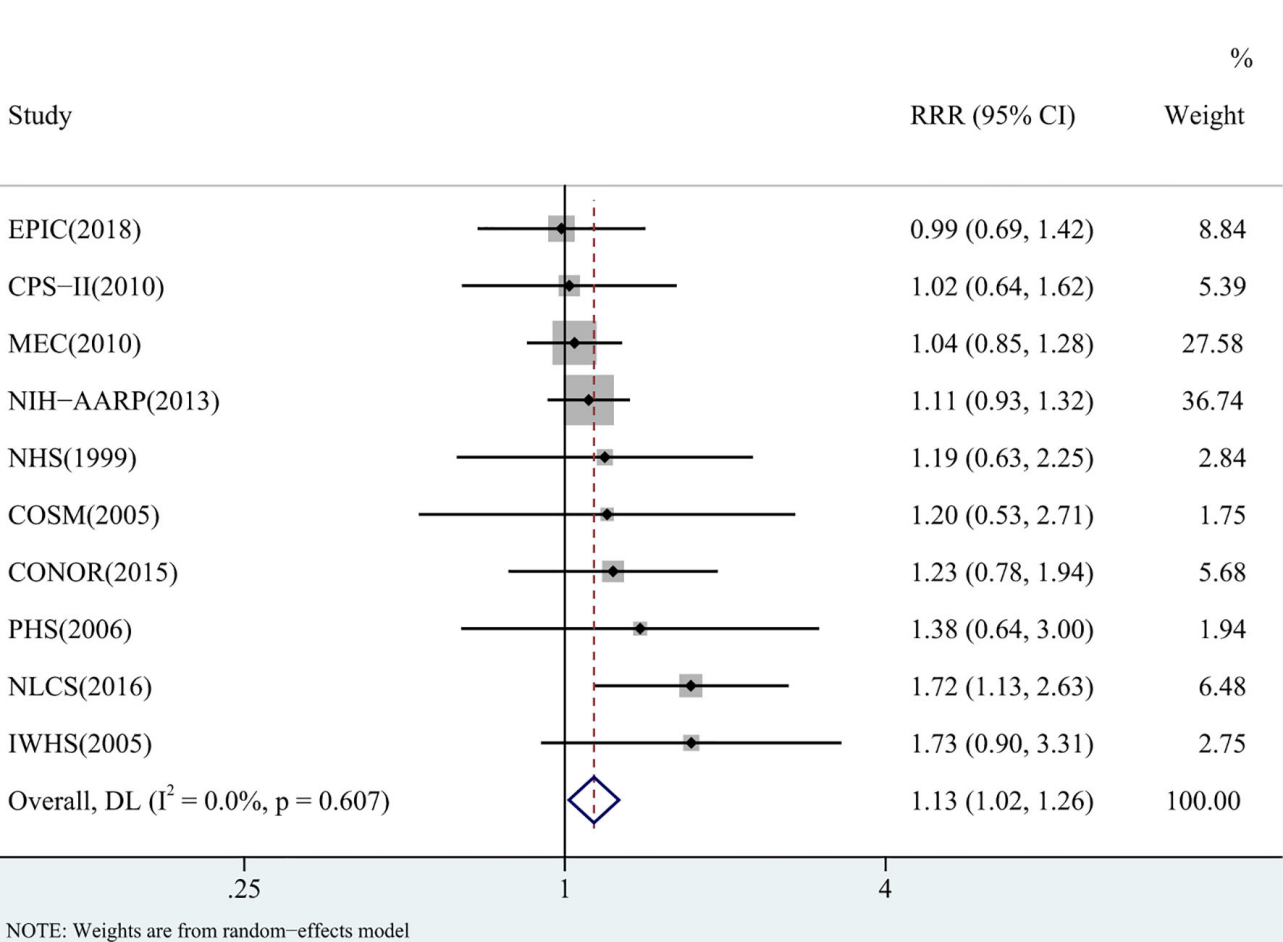
Pooled RCC-to-LCC ratio of relative risks (RRRs) for comparing the association between diabetes and the risk of right-sided colon cancer (RCC) with that of left-sided colon cancer (LCC).

The pooled RR for diabetes was significantly higher in RCC than in LCC (RRR = 1.13, 95% CI = 1.02-1.26) (**Figure 3**). There was no heterogeneity between studies (I^2^ = 0%, p = 0.607) and no indication of publication bias (Begg’s test: p = 0.283, Egger’s test: p = 0.139; **Supplementary Figure S3**). In the sensitivity analyses, the pooled RRRs did not differ significantly by geographical area (p = 0.406), sex (p = 0.430), definition of RCC and LCC (p = 0.411), duration of follow-up (p = 0.171) or the level of adjustment (p = 0.837) (**Table 3**).

**Table 3:** RCC-to-LCC ratio of relative risks (RRRs) and 95% confidence intervals (CIs) for sensitivity analysis.

	No. of studies	RRR (95%CI)	I^2^,%	*P*_heterogeneity_	*P* value for interaction
**Geographical area**					
USA	6	1.10 (0.98-1.25)	0	0.758	0.406
Europe	4	1.25 (0.96-1.63)	22.7	0.275	
**Sex**					
Males	5	1.12 (0.95-1.33)	0	0.824	0.430
Females	5	1.26 (1.00-1.58)	0	0.456	
**Definition of RCC and LCC**					
Definition 1	8	1.10 (0.95-1.27)	0	0.860	0.411
Definition 2	2	1.32 (0.87-2.02)	71.9	0.059	
**Duration of follow-up**					
< 15 years	5	1.09 (0.96-1.23)	0	0.645	0.171
≥ 15 years	5	1.30 (1.04-1.64)	0	0.572	
**Level of adjustment**					
Age-adjusted	5	1.04 (0.89-1.23)	0	0.932	0.837
Multi-adjusted	5	1.07 (0.90-1.27)	0	0.950	

## Discussion

In this pooled analysis of prospective cohort studies, with data from 1,642,823 individuals and 17,624 CC events, we examined the site-specific association between diabetes and the risk of CC. Our findings support a role for diabetes in the etiology of CC, including both RCC and LCC. After adjusting for major risk factors, individuals with diabetes showed a significant 13% increased risk of RCC compared with LCC.

The main strength of this pooled analysis is that by including only prospective cohort studies, we were able to perform an objective meta-analysis based on a large population size over a long follow-up duration. The ten large prospective cohort studies included better resemble clinical practice, are of good quality and lack obvious selection bias compared with case-control studies and retrospective cohorts. Our findings were robust with no heterogeneity across studies and no obvious evidence of publications bias. Moreover, our results were consistent in a range of subgroup analyses.

Diabetes and CC share similar risk factors, including obesity, physical activity and the Western diet. Thus, our results could be confounded by these risk factors. However, a previous meta-analysis found that BMI and meat consumption were associated with a more pronounced risk of LCC, while physical activity and other dietary risk factors did not differ between LCC and RCC (39–41). In our analysis, nine out of ten studies adjusted for major risk factors (≥ 5, including obesity, smoking and physical activity) of CC in addition to age. Importantly, there was no evidence of a significant difference between age-adjusted RR/RRRs and multi-adjusted RR/RRRs. Thus, it is unlikely that the results were influenced by confounders. In addition, we directly compared the risk of RCC and LCC from within the same study, thereby reducing the role of extraneous, between-study known and unknown confounding factors.

Nevertheless, several limitations merit comment. First, there are racial/ethnic differences in the incidence of CRC. According to the 2014 Surveillance, Epidemiology, and End Results (SEER) Cancer Statistics, CRC incidence rates were highest among blacks, followed by American Indians/Alaska Natives, non-Hispanic whites, Hispanics and Asian/Pacific Islanders (42). In our study, except for the multiethnic cohort, the patients included were predominantly white from the other nine cohorts. Further studies are warranted to address whether this increased risk of RCC compared with that of LCC remains in other races/ethnicities. Second, diabetes status was based on self-report, which could have resulted in some misclassification of diabetics as nondiabetics. Although previous studies have shown that self-reported diabetes is an accurate proxy compared with medical records (43, 44), such misclassification still could underestimate the true relationship between diabetes and CC. Third, the studies included in this analysis did not distinguish between type 1 and type 2 diabetes. As type 1 diabetes accounts for 5-10% of all cases of diabetes and may not be related to CC (45), associations were likely to be underestimated in this study. Nevertheless, the incidence of type 1 diabetes increases with age, peaking at around 10–14 years (46). The greatest observed increases in the incidence of type 1 diabetes are among children younger than 15 years, particularly those younger than 5 years (47). In our study, all patients enrolled were ≥ 30 years old, thus they were much less likely to have type 1 diabetes than type 2 diabetes. Fourth, not all of the CC cases could be classified by location, which may have influenced the real RRs or RRRs. In our analysis, we only included studies which classified at least 80% of CRC cases or CC cases by subsite. Fifth, most of the studies involved in this analysis did not report on, or adjust for antidiabetic drug use, which could influence the true associations between diabetes and CC (48). However, the relationship between the use of anti-diabetic drugs and the incidence of colon cancer is still unclear (49–52). Previous meta-analyses suggested that metformin potentially reduces the risk of colon cancer in patients with type 2 diabetes (53–55), while insulin use was associated with an increased risk (50, 51). Some preclinical studies also showed that the SGLT2 inhibitors might attenuate colon cancer cells growth (56–59).

The hyperinsulinemia theory, which implies that elevated insulin and free IGF-1 levels are the two key components that promote the growth of colon tumors (60), may relate diabetes to colon cancer. Insulin and IGF-1 receptors are widely distributed in normal colonic epithelium and colon cancer tissue (61, 62). Insulin is an important growth factor for colonic mucosal cells (63). Preclinical studies have shown that insulin promotes not only the growth and survival of colon cancer cells but also the biosynthesis of IGF-I (64), while IGF-1 inhibits the apoptosis of colon cancer cells (65). There is some potential biologic evidence to explain why diabetes may have different associations with the risks of RCC and LCC. Leptin, which is regulated by insulin (66), has been shown to increase colonic cell proliferation and stimulate DNA synthesis in the proximal colon, but not in the distal colon (67). In an IGF-1-deficient rodent model, reduction of IGF-1 altered the location of the colonic tumor. Significant inhibition of colon tumor multiplicity in the proximal colon was observed compared to that in the distal colon (68). The distinct sensitivity to leptin or IGF-1 between proximal and distal colon cells implies differences in genetic susceptibilities to carcinogens through different molecular mechanisms.

In 2014, the worldwide prevalence of type 2 diabetes was approximately 422 million, accounting for 8.4% of adults, and this rate is projected to increase rapidly. In 2019, diabetes was the direct cause of 1.5 million deaths (69). The present study provides the most comprehensive assessment of possible site differences between diabetes and CC risk. Our findings may have important clinical implications for customized CRC screening programs in diabetic patients. CRC screening tests using FS or colonoscopy are now common in many countries and have been proven effective to reduce CRC incidence and mortality. Although FS is more widely available, quicker, more convenient and less expensive than colonoscopy, it allows visualization of only the distal part of the colon, while colonoscopy allows visualization of the entire colon (10, 70). Hence, individuals who are at high risk for proximal lesions, such as diabetic patients, are more suited for colonoscopy; FS may be of less value. Our findings reinforce the importance of stratifying for high-risk populations, such as patients with diabetes to improve the effectiveness of CRC screening and prevention. Close colonoscopic surveillance in diabetic patients with careful examination of the right colon is warranted.

To conclude, the results from this meta-analysis suggest that diabetes is associated with increased risks for both RCC and LCC and that the association between diabetes and the risk of RCC is significantly higher than the risk of LCC after adjusting for major risk factors. Our findings add to the increasing evidence for the distinct clinical and biological patterns between RCC and LCC and provide further support for public health efforts aiming to establish a more tailored approach to CRC screening strategies in diabetic patients.

## Data Availability Statement

The original contributions presented in the study are included in the article/**Supplementary Material**. Further inquiries can be directed to the corresponding author.

## Author Contributions

WX and BW contributed to the study concept and design, data analysis, interpretation of the data and editing the manuscript. WX and JH contributed to the data acquisition, statistical analysis and editing the manuscript. WX, JH, CZ and LD contributed to the statistical analysis. WX, JH and XW contributed to the interpretation of the data. All authors commented on drafts of the paper and have approved the final draft of the manuscript for submission.

## Conflict of Interest

The authors declare that the research was conducted in the absence of any commercial or financial relationships that could be construed as a potential conflict of interest.

## Publisher’s Note

All claims expressed in this article are solely those of the authors and do not necessarily represent those of their affiliated organizations, or those of the publisher, the editors and the reviewers. Any product that may be evaluated in this article, or claim that may be made by its manufacturer, is not guaranteed or endorsed by the publisher.
