# Supplementary material for: Diabetes and Risks of Right-Sided and Left-Sided Colon Cancer: A Meta-Analysis of Prospective Cohorts
Source: Front Oncol. 2022 Apr 7;12:737330. doi: 10.3389/fonc.2022.737330 (PMC9021717; doi:10.3389/fonc.2022.737330)
Supplement: Supplementary file 1 [file DataSheet_1.docx]

**Title:** **Diabetes and risks of right-sided and left-sided colon cancer: A meta-analysis of prospective cohorts**

**Authors:** Wenxuan Xiao^1†^, Jinglong Huang^1†^, Chuanyi Zhao^1^, Lu Ding^1^, Xuan Wang^2^, Bian Wu^1*^

^1^Cancer Center, Union Hospital, Tongji Medical College, Huazhong University of Science and Technology, Wuhan 430022, China.

^2^Department of Neurosurgery, Union Hospital, Tongji Medical College, Huazhong University of Science and Technology, Wuhan, Hubei, China.

†Wenxuan Xiao and Jinglong Huang contributed equally to this research.

*Correspondence to Bian Wu (e-mail: [bian.wu@outlook.com](mailto:bian.wu@outlook.com))

**Supplementary Methods**

**Search Strategy**

The Pubmed search was performed using the following search strategy: ("diabetes mellitus"[Mesh] OR “diabetes mellitus” OR “glucose intolerance” OR “insulin resistance” OR “DM” OR “diabetic” OR “diabetes”) AND (“colorectal” OR “bowel” OR “rectum” OR “colorectum” OR “colon” OR “rectal”) AND ("neoplasms"[Mesh] OR "carcinoma"[Mesh] OR “neoplasm” OR “tumour” OR “carcinoma” OR “tumor” OR “cancer” OR “neoplasia”).

The Embase search was performed using the following search strategy: (“diabetes mellitus”/exp OR “diabetes mellitus” OR “glucose intolerance” OR “insulin resistance” OR “DM” OR “diabetic” OR “diabetes”) AND (“colorectal” OR “bowel” OR “rectum” OR “colorectum” OR “colon” OR “rectal”) AND (“neoplasms”/exp OR “Carcinoma”/exp OR “neoplasm” OR “tumour” OR “carcinoma” OR “tumor” OR “cancer” OR “neoplasia”).

The Web of Science search was performed using the following search strategy: (“diabetes mellitus” OR “glucose intolerance” OR “insulin resistance” OR “DM” OR “diabetic” OR “diabetes”) AND (“colorectal” OR “bowel” OR “rectum” OR “colorectum” OR “colon” OR “rectal”) AND (“neoplasm” OR “tumour” OR “carcinoma” OR “tumor” OR “cancer” OR “neoplasia”).

The Cochrane Library database search was performed using the following search strategy: ("diabetes mellitus"[Mesh] OR “diabetes mellitus” OR “glucose intolerance” OR “insulin resistance” OR “DM” OR “diabetic” OR “diabetes”) AND (“colorectal” OR “bowel” OR “rectum” OR “colorectum” OR “colon” OR “rectal”) AND ("neoplasms"[Mesh] OR "carcinoma"[Mesh] OR “neoplasm” OR “tumour” OR “carcinoma” OR “tumor” OR “cancer” OR “neoplasia”)

**Supplementary Figures**

**Supplementary Figure S1:** Funnel plot of the pooled relative risks (RRs) for the association between diabetes and the risks of RCC.

**
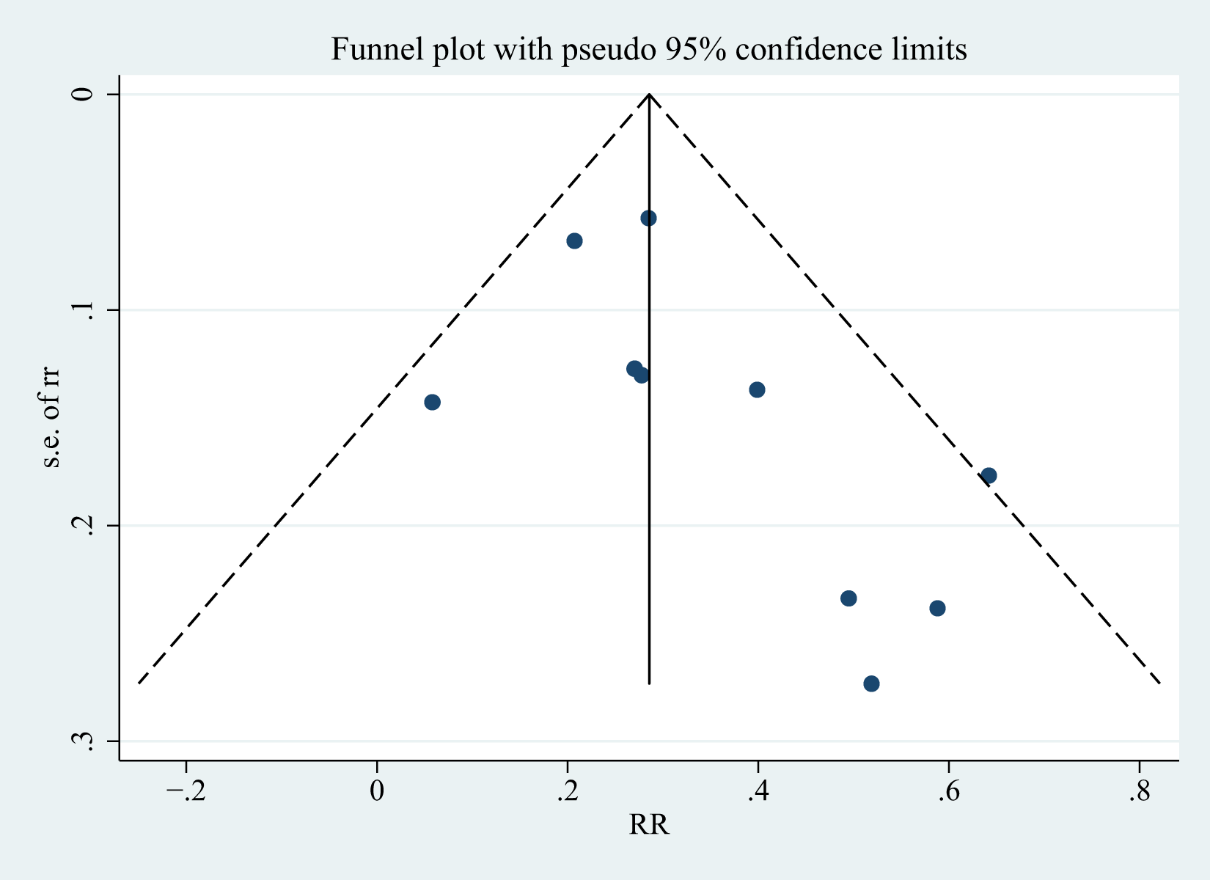
**

**Supplementary Figure S2:** Funnel plot of the pooled relative risks (RRs) for the association between diabetes and the risks of LCC.

**
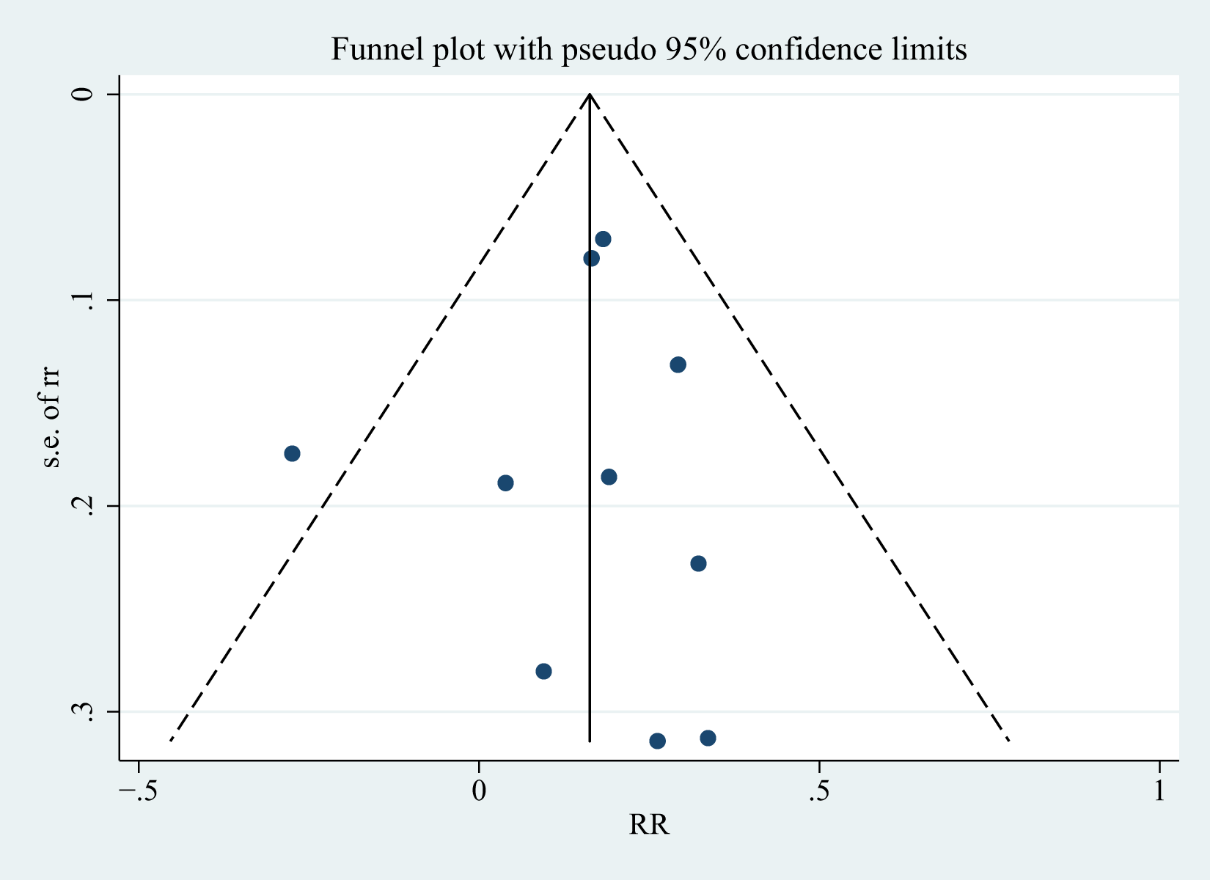
**

**Supplementary Figure S3:** Funnel plot of the pooled RCC-to-LCC ratio of relative risks (RRRs) for comparing the association between diabetes and the risk of RCC with that of LCC.


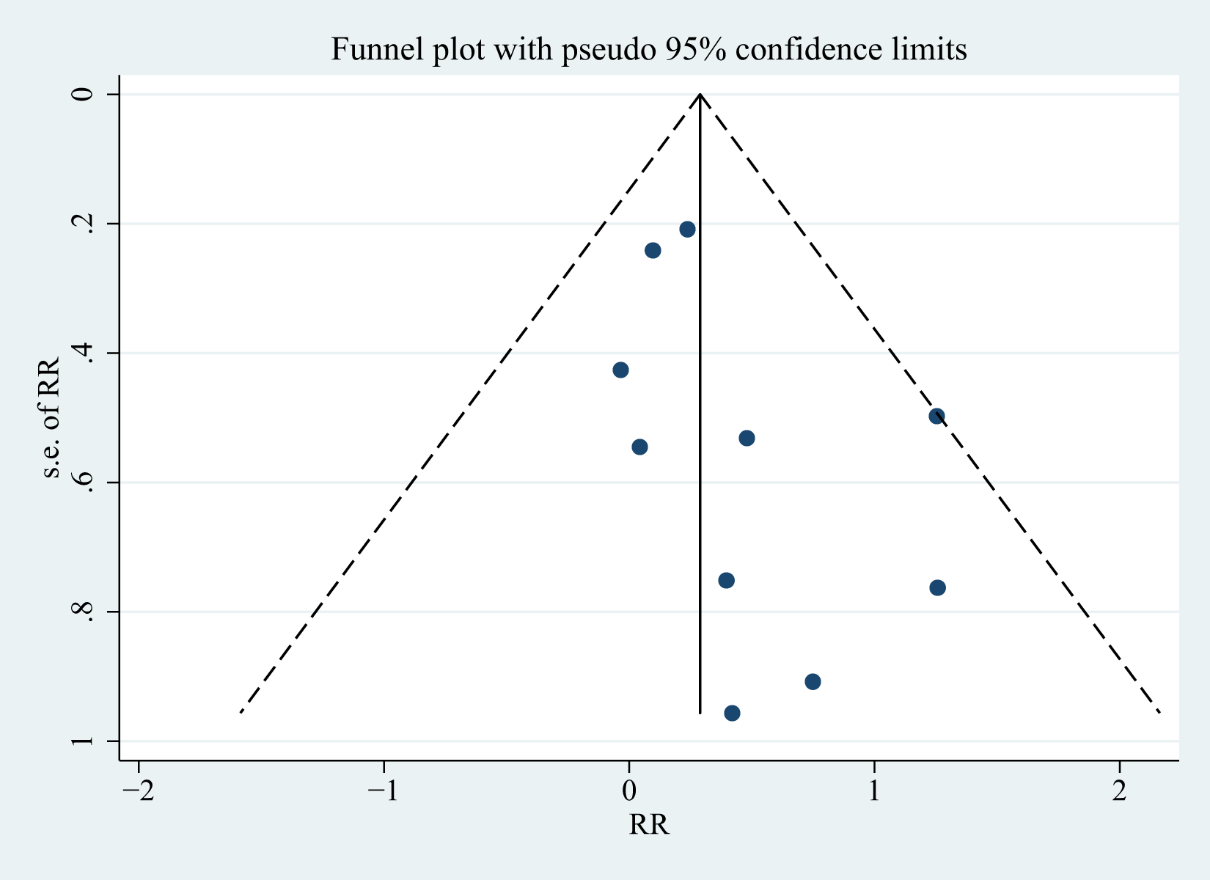


**Supplementary Tables**

**Supplementary Table S1:** Methodological quality assessment of included studies based on the Newcastle-Ottawa Scale

| **Study** | **Year** | **Selection** | | | | **Comparability** | **Outcome** | | | **Total** |
| --- | --- | --- | --- | --- | --- | --- | --- | --- | --- | --- |
|  |  | **Representativeness of the exposed cohort** | **Selection of the non exposed cohort** | **Ascertainment of exposure** | **Outcome of interest was not present at start of study** | **Control for important factors^a^** | **Assessment of outcome** | **Follow-up long enough for outcomes to occur^b^** | **Adequacy of follow-up of cohorts^c^** |  |
| NHS | 1999 | 0 | 1 | 0 | 1 | 2 | 1 | 1 | 1 | 7 |
| COSM | 2005 | 1 | 1 | 0 | 1 | 2 | 1 | 0 | 1 | 7 |
| IWHS | 2005 | 0 | 1 | 0 | 1 | 2 | 1 | 1 | 1 | 7 |
| PHS | 2006 | 0 | 1 | 0 | 1 | 2 | 1 | 1 | 1 | 7 |
| CPS-II | 2010 | 1 | 1 | 0 | 1 | 2 | 1 | 1 | 1 | 8 |
| MEC | 2010 | 1 | 1 | 0 | 1 | 2 | 1 | 1 | 0 | 7 |
| NIH-AARP | 2013 | 1 | 1 | 0 | 1 | 2 | 1 | 1 | 1 | 8 |
| CONOR | 2015 | 1 | 1 | 0 | 1 | 2 | 1 | 1 | 0 | 7 |
| NLCS | 2016 | 1 | 1 | 0 | 1 | 1 | 1 | 1 | 1 | 7 |
| EPIC | 2018 | 1 | 1 | 0 | 1 | 2 | 1 | 1 | 1 | 8 |

^a^A maximum of 2 stars could be awarded for this item. Studies that controlled for age received one star, whereas studies that controlled for other factors (body mass index, physical activity or smoking) received an additional star.

^b^A cohort study with a follow-up time > 5 years was assigned one star.

^c^A cohort study with a follow-up rate > 80% was assigned one star.
